# Deep learning and multi-omics reveal programmed cell death-associated diagnostic signatures and prognostic biomarkers in gastric cancer

**DOI:** 10.3389/fimmu.2025.1690200

**Published:** 2025-11-10

**Authors:** Qiaoying Jin, Zhaobin Chang, Kangping Chen, Na Jiang, Guoxiu Chen, Yonggang Lu

**Affiliations:** 1 School of Information Science and Engineering, Lanzhou University, Lanzhou, China; 2 The Second Hospital & Clinical Medical School, Lanzhou University, Lanzhou, China; 3 School of Computer Science and Artificial Intelligence, Lanzhou University of Technology, Lanzhou, China

**Keywords:** deep learning, multi-omics, diagnosis and prognostic, biomarkers, gastric cancer

## Abstract

Gastric cancer (GC) is characterized by pronounced molecular and clinical heterogeneity, creating major challenges for therapeutic decision-making. Limitations in current molecular classification hinder the development of personalized therapies, underscoring the need for improved diagnostic and prognostic frameworks. we conducted an integrated multi-omics analysis of bulk, single-cell, and spatial transcriptomic data to systematically characterize three key programmed cell death pathways—pyroptosis, apoptosis, and necroptosis (collectively abbreviated as PAN). A scoring-based clustering framework integrating multiple machine learning algorithms was developed to define high-resolution molecular subtypes and construct a deep learning signature. A hybrid CNN+BiLSTM model with cross-fusion attention was applied for transcriptomic feature extraction and subtype classification, achieving superior performance compared with existing approaches. Validation in the TCGA cohort confirmed the robustness and biological relevance of our model. Among the identified subtypes, Subtype 2 showed the most favorable prognosis. We further established a nine-gene prognostic signature with strong predictive value. High-risk patients exhibited poor survival, enhanced immune infiltration, and potential sensitivity to AKT inhibitors, with several drugs, including gefitinib and paclitaxel, identified as promising candidates. Experimental validation was conducted using the Human Protein Atlas (HPA) and RT-qPCR in clinical samples. CFLAR and TNFSF13B were upregulated and PDK4 downregulated in GC, while UACA showed no significant change. Additional prognostic genes (DFFB, PSMB6, GLP1R, HDAC9, BACH2) displayed expression patterns largely consistent across HPA, TCGA, and RT-qPCR, with minor discrepancies likely due to sample size. This study integrates multi-omics and deep learning with experimental validation, providing insights into programmed cell death regulation and offering robust biomarkers and therapeutic targets for GC.

## 1 Introduction

Gastric cancer remains a major contributor to cancer-related deaths globally, with especially high incidence rates in East Asia (1, 2). Despite ongoing improvements in clinical management, advanced gastric cancer continues to be associated with a poor prognosis, highlighting the urgent need to understand its underlying molecular mechanisms (3). Gastric cancer exhibits remarkable biological complexity characterized by sustained inflammatory signaling, immune evasion, and metabolic reprogramming (4–6). These factors contribute to tumor heterogeneity and therapeutic resistance, necessitating a more precise stratification of patients and personalized treatment strategies.

Recent research has spotlighted the role of programmed cell death (PCD) pathways including PAN in shaping the tumor immune microenvironment and modulating tumor progression (7, 8). Each PCD pathway contributes uniquely to immune surveillance and inflammation. For example, pyroptosis triggers a potent inflammatory response that may enhance tumor immunogenicity (9), whereas necroptosis and apoptosis exert context-dependent effects on tumor immunity (10). Moreover, increasing evidence suggests that these PCD modes are not mutually exclusive but instead interconnect through shared molecular components, forming an intricate cell death regulatory network (11–13).

Despite growing interest in PAN, several critical knowledge gaps remain, particularly in the context of gastric cancer. A major limitation lies in the absence of comprehensive analyses that integrate multiple layers of transcriptomic data, including bulk RNA-seq, single-cell RNA-seq (scRNA-seq), and spatial transcriptomics—to systematically dissect the molecular landscape of PAN in gastric tumors (14–16). Most existing studies are confined to single-omic platforms and thus fail to capture the complex regulatory networks and cell-type-specific features underlying. Furthermore, PAN-related prognostic signatures and their associations with the tumor microenvironment (TME) have not been fully elucidated (17), and clinically applicable risk models are still lacking. These limitations hinder the translation of mechanistic insights into practical tools for clinical decision-making. Addressing these gaps requires a systems biology framework that leverages high-throughput datasets and advanced computational strategies to uncover robust biomarkers and predictive models for gastric cancer.

To address these challenges, we employed a comprehensive multi-omics strategy integrating machine learning and transcriptomic analysis to characterize PAN-related biomarkers in gastric cancer. Specifically, to identify core PAN-related genes, we applied both classical clustering algorithms (e.g., K-means, Gaussian Mixture Model) and deep learning approaches (Convolutional Neural Network (CNN)+Bidirectional Long Short-Term Memory (BiLSTM) with cross-fusion attention) to bulk RNA-seq data. These findings were further validated through single-cell and spatial transcriptomics, providing cellular and spatial resolution of PAN activity. We constructed an innovative prognostic model, PANscore, by integrating Cox regression with a RSF algorithm, which effectively stratifies patient prognosis and elucidates the immune infiltration, and drug sensitivity of PAN, offering a foundation for personalized therapeutic strategies in gastric cancer and advancing the goals of precision oncology.

## 2 Materials and methods

### 2.1 Data acquisition and processing

The Cancer Genome Atlas (TCGA, https://portal.gdc.cancer.gov/) provides both clinical annotations and bulk RNA-seq datasets for gastric cancer patients analyzed in this study, as well as corresponding data for other cancer types such as breast cancer (BRCA) and cervical cancer (CESC). To ensure data reliability, samples with incomplete clinical information were excluded, resulting in a final dataset comprising 375 gastric cancer samples and 73 independent healthy control samples. For external validation, test datasets were downloaded from the Gene Expression Omnibus (GEO, https://www.ncbi.nlm.nih.gov/geo/) database with the accession IDs GSE62254 and GSE66229. ScRNA-seq data and spatial transcriptomics data were obtained from GSE183904 and GSE251950, respectively.

### 2.2 Bulk RNA-seq data were analyzed to identify PAN-related differentially expressed genes

The PAN gene set was constructed by systematically integrating gene lists associated with PAN, obtained from multiple curated pathway databases, including Reactome, AmiGO 2, and KEGG. To ensure specificity, redundant genes shared across multiple cell death pathways were identified and removed, resulting in a non-overlapping, high-confidence PAN-related gene set that reflects the distinct yet interconnected nature of these programmed cell death modalities.

### 2.3 A scoring-based clustering algorithm was implemented to analyze PAN-related gene expression patterns in bulk transcriptomic

**Figure 1:**
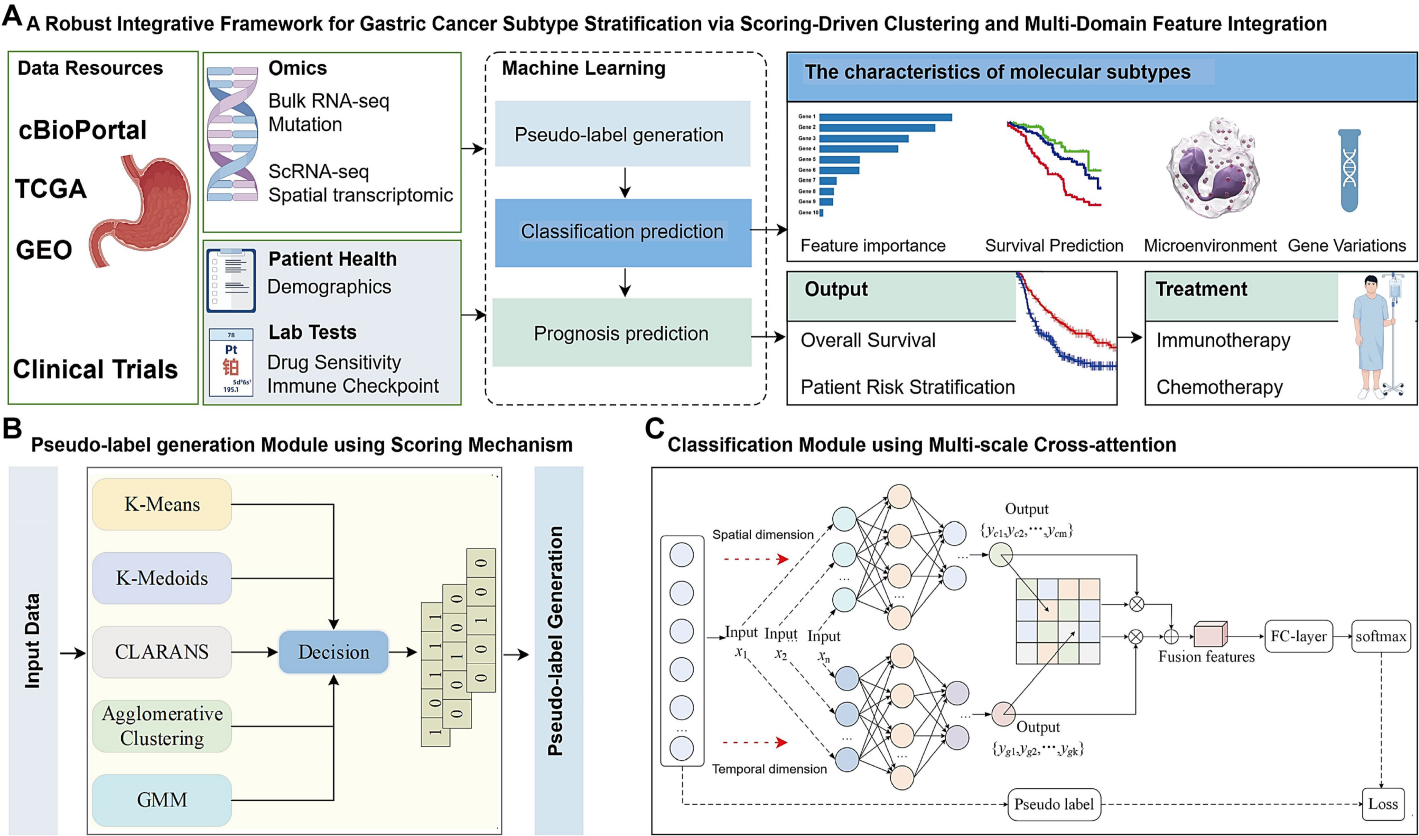
Framework overview. **(A)** The model integrates scoring-based clustering and multi-domain feature fusion to convert complex data into actionable insights. The framework operates as a deep learning model to stratify patient subtypes. Prognosis predictions also established by machine learning. **(B)** Pseudo-label generation Module using Scoring Mechanism in self-supervised mode. Input data are represented by [Key, Value] pairs, where the key is the feature name and the value corresponds to the numerical score of the feature. Feature names and values are embedded and fed to a Scoring Clustering architecture without positional encoding. The output of this scoring clustering is Pseudo label. **(C)** Classification Module using Multi-scale Cross-attention, for a given gene sequence X, the sequence X is initially fed into a Convolutional Neural Network (CNN) and a Bidirectional Long Shot-Term Memory (BiLSTM) to extract coding features from the spatial and temporal dimensions. Respectively. Subsequently, a cross-attention mechanism is employed to align and facilitate information interaction between the spatial and temporal features, thereby fusing the features across different dimensions, this fusion aims to construct a classifier based on the integrated feature representation. Finally, the fused features are input into a fully connected (FC) laver to adjust their dimensionality and the classification is performed using a SoftMax function.

We developed an integrative framework that combines ensemble clustering and deep learning to predict molecular subtypes of genes. Initially, bulk RNA-seq datasets were split into training and test sets, and pseudo-labels were generated using a voting-based scoring network that integrates five clustering algorithms (K-Means, GMM (Gaussian Mixture Model), Agglomerative Clustering, CLARANS (Clustering Large Applications based on RANdomized Search), and K-Medoids). To enhance reliability, the pseudo-labels were biologically validated through analyses of immune infiltration patterns, checkpoint molecule expression, and drug response characteristics. These labels were then used to train a classification model featuring a parallel cross-fusion attention architecture, which leverages CNNs for spatial information and BiLSTM for capturing temporal features. The model achieved robust subtype classification performance, as shown in **Figure 1**.

#### 2.3.1 Model architecture

The overall workflow for predicting different gene subtypes using a scoring network constructed by five clustering algorithms is shown in **Figure 1B**. Initially, training and test sets are constructed using bulk RNA-seq datasets, with specific genes, such as X1 and X2, among Y gene types selected to validate the reliability of the clustering pseudo-label generation phase and prediction phase of the designed scoring network. Subsequently, an integrated scoring network is designed by combining K-Means, GMM, Agglomerative Clustering, CLARANS, and K-Medoids clustering algorithms. Pseudo-labels are assigned to each gene using a majority voting mechanism. The reliability of the clustering phase’s pseudo-labels was further validated using biological assays such as immune infiltrating, immune checkpoint genes expression level, chemotherapy sensitivity. Finally, a parallel cross-fusion network was constructed, incorporating BiLSTM for sequence modeling and CNN for feature extraction. The model was trained using the dataset with pseudo-labels to achieve classification of different gene subtypes (**Figure 1A**).

#### 2.3.2 Pseudo-label generation module

The TCGA and GEO datasets do not provide true label information. Existing molecular typing methods typically use clustering algorithms, such as K-Means or Consensus Clustering (CC), to cluster and reorganize TCGA or GEO data, generating pseudo-labels for gene sequences, which are then used to train supervised classification models. However, the reliability of pseudo-labels generated by a single clustering algorithm remains an open question. It is well known that the reliability of pseudo-labels during the clustering phase directly impacts the prediction accuracy of classification models.

To address this, we developed a novel scoring network using K-Means, GMM, Agglomerative Clustering (CLARA, Clustering LARge Applications), CLARANS, and K-Medoids (PAM, Partitioning Around Medoids) clustering algorithms. In this network, each gene is assigned a corresponding pseudo-label via a majority voting mechanism. Specifically, our scoring network integrates the strengths of different clustering algorithms, measuring the distribution differences across various datasets from multiple perspectives. This approach is better suited to adapt to the complex distributions across different data domains, influenced by factors such as environment, individual variability, and drug sensitivity.

Notably, to enhance the stability of clustering results and reduce ambiguity between data domains, our scoring network standardizes the distance metric strategies of DBSCAN, K-Medoids, and Agglomerative Clustering by uniformly employing the Euclidean distance metric. This standardization ensures consistent distribution differences across algorithms, both between different sample clusters and within data points in the same cluster (**Figure 1B**).

#### 2.3.3 CNN+ BiLSTM parallel cross-fusion attention classification model

In existing gene sequence prediction methods, machine learning algorithms such as decision trees, Bayesian classifiers and Support Vector Machines (SVM) are commonly employed to construct classification models. However, limited attention has been paid to the construction of classifiers, particularly ensemble classifiers based on deep learning models. High-quality classifiers are crucial for improving prediction accuracy. To this end, this study proposes a novel ensemble classification model leveraging CNN and BiLSTM to extract both temporal and spatial encoding information from gene sequence data. The extracted multi-scale features are then employed in building the classification model, as shown in **Figure 1C**.

For a given gene sequence $X=\left\{x_{1},x_{2},\ldots ,x_{n}\right\}$, the gene sequence is used as input to both the CNN and the BiLSTM. These models extract encoding features from the gene sequence in both the spatial and temporal dimensions, as illustrated by the calculation in Equation 1:

(1) $$\{\begin{array}{c} Fc=w_{i}\left(x_{1},x_{2},\ldots ,x_{n}\right)+b_{i}\\ Fg=w_{j}\left(x_{1},x_{2},\ldots ,x_{n}\right)+b_{j} \end{array}$$

Here, Fc and Fg represent the encoding features in the spatial and temporal dimensions, respectively. In this context, a neural network with three convolutional layers is used to extract the encoding features in the spatial dimension. $w_{i}$ and $w_{j}$ denote the weights, which are a set of learnable parameters, and $b_{i}$ and $b_{j}$ represent the biases.

To facilitate the alignment and information exchange between features from different dimensions, a cross-attention mechanism is employed to establish interactions between the temporal and spatial encoding features. The goal is to fuse these encoding features and use the fused features to construct the classifier. The feature fusion computation between dimensions is given by Equation 2:

(2) $$Fcg=\text{softmax}\left(\frac{Fc\cdot Fg^{\text{T}}}{\sqrt{d}}\right)\left(Fc+Fg\right)$$

Here, Fcg denotes the fused features, which encapsulate both the temporal and spatial dimensional encoding semantics. d represents the feature dimension, and T denotes the transpose operation.

Finally, the fused encoding features from both the temporal and spatial dimensions are used as input to a fully connected layer (FC-layer), where the dimensionality of the fused features is adjusted. Softmax is used as the classification function. To optimize the performance of the temporal-spatial parallel fusion classification model, a cross-entropy loss function is used to compute the loss between the predicted labels $\text{P}=\left\{{\text{p}}_{1},{\text{p}}_{2},\ldots ,{\text{p}}_{k}\right\}$ and the clustering pseudo-labels $\text{Y}=\left\{{\text{y}}_{1},{\text{y}}_{2},\ldots ,{\text{y}}_{k}\right\}$. The reliability of the clustering pseudo-labels is further validated through subsequent biological analyses.

### 2.4 Screening of optimal PAN-related gene

To identify gastric cancer specific signature genes, LASSO and random forest algorithms were independently applied, leveraging their complementary advantages in feature selection efficiency and model stability (18, 19). LASSO regression was conducted via the “glmnet” package, introducing an L1 regularization term to constrain model complexity and facilitate the selection of informative features by penalizing less relevant or redundant genes. Concurrently, Random Forest, through the construction and aggregation of multiple decision trees, was employed to evaluate feature importance and identify the most discriminative gene candidates. The overlapping genes derived from these two machine learning approaches were subsequently defined as hub PAN-related genes in gastric cancer.

### 2.5 Data collection of somatic variants and copy number variants

Given its correlation with neoantigen load, Tumor mutational burden (TMB) has become a widely used metric for assessing potential benefit from immunotherapy. To assess its clinical relevance, we conducted stratified survival analyses to examine the association between TMB and patient prognosis. Somatic mutation profiling was performed using the “maftools” R package (20). The analysis identified the top 30 genes with the highest mutation frequencies, offering insights into mutation patterns associated with gastric cancer subgroups. The copy number variation landscape was comprehensively analyzed using the “DNAcopy” and “VariantAnnotation” R packages.

### 2.6 Immune cell infiltration

We utilized a comprehensive suite of bioinformatics tools—including quanTIseq, ESTIMATE, MCPcounter, single-sample Gene Set Enrichment Analysis (ssGSEA) and xCell—to assess immune status differences among the molecular subtypes (21–24). These approaches enabled a detailed characterization of immune cell infiltration within the TME. Furthermore, we investigated the associations between molecular subtypes and immune checkpoint gene expression to elucidate potential implications for immunotherapeutic responsiveness (25, 26).

### 2.7 Single-cell RNA-seq analysis and cell-cell interaction mapping of PAN-related gene in gastric cancer

To elucidate the cellular heterogeneity and regulatory networks in gastric cancer, we conducted a comprehensive scRNA-seq analysis using the dataset from GSE183904. Our analysis employed the “Seurat” and “SingleR” R packages, following a series of standardized quality control procedures. Low-quality cells were filtered out using the “PercentageFeatureSet” function based on mitochondrial gene percentage and unique molecular identifier (UMI) counts. The remaining cells were processed using the “SCTransform” function for normalization, variance stabilization, and removal of technical artifacts. The “RunPCA” function was employed to perform principal component analysis (PCA) on highly variable genes for dimensionality reduction. Cell clusters were identified based on transcriptional profiles using the “FindNeighbors” and “FindClusters” functions, and annotation of cell types was performed using the “SingleR” functions, supplemented by well-characterized marker genes from previous studies (27). To reconstruct cellular developmental trajectories, we performed pseudotemporal ordering using the Monocle package. We delineated distinct cell populations through clustering and constructed a single-cell expression matrix to elucidate the developmental states of cells. By analyzing dynamic gene expression patterns, we inferred the differentiation trajectories of cells. We employed the CytoTRACE algorithm to quantitatively evaluate the developmental potential of individual cells (28, 29). Concurrently, we utilized CellChat to investigate cell-cell communication networks (30). By leveraging its integrated CellChatDB ligand-receptor interaction database, we systematically identified cell type-specific signaling patterns and characterized the dynamics of intercellular communication. This approach detected significantly overexpressed ligands and receptors within distinct cell populations, enabling the inference of enhanced intercellular signaling pathways.

To further investigate gastric cancer at single-cell resolution, we employed the SCENIC pipeline (31) for gene regulatory network inference and validated key gene expression patterns across different cell populations using dataset GSE183904. Our analytical workflow included: (1) stringent quality control by excluding cells with >25% mitochondrial gene content or extreme gene counts (<500 or >6000 genes); (2) data normalization using Seurat’s “NormalizeData” function with default parameters; (3) expression matrix standardization via “ScaleData”; and (4) dimensionality reduction through PCA was applied to the top 3,000 highly variable genes for dimensionality reduction, followed by t-SNE visualization (using 30 principal components) implemented with the “RunTSNE” function.

### 2.8 Processing and analysis of the spatial transcriptome data

To characterize the spatial gene expression architecture in gastric cancer, we analyzed spatial transcriptomics data (GSE251950) from GEO, comprising filtered feature-barcode matrices and corresponding spatial coordinates. The data processing pipeline included normalization and scaling using SCTransform (Seurat v4.0) (32), followed by dimensionality reduction via PCA (RunPCA) and t-SNE visualization (top 30 PCs) to reveal global expression patterns. We then performed clustering (resolution=0.5) to identify spatially coherent transcriptomic domains. For cellular resolution analysis, we implemented the Spacexr package (33) to deconvolute Visium spots, first creating reference profiles (create.RCTD) and then executing deconvolution (run.RCTD) in full mode to estimate the cellular composition of each spot, thereby enabling comprehensive mapping of cell type distributions within the tissue architecture.

### 2.9 RSF-driven prognostic model construction and assessment

First, univariate Cox regression analysis was performed to identify survival-associated genes based on gene expression levels. Subsequently, a RSF (34) model was constructed using the selected genes, and the importance of each gene was ranked according to its contribution to the model’s predictive performance. The model’s predictive performance was evaluated over time using the Concordance Index (C-index). As the ensemble of decision trees expanded, the model’s prediction error progressively diminished and ultimately reached a stable plateau, underscoring the model’s robustness and reliability. Key variables with significant impacts on survival prediction were further selected based on their importance scores. Patients were stratified into high-risk and low-risk groups based on the risk scores derived from the RSF model. This stratification was achieved using maximally selected rank statistics, which identified the optimal cutoff value to maximize the survival differences between the two groups. Kaplan-Meier analysis revealed significant survival disparity between these risk strata. The prognostic robustness of this classification was subsequently confirmed in an independent validation cohort, demonstrating consistent survival trends across datasets.

### 2.10 Chemotherapeutic response profiling

To evaluate differential drug sensitivity between risk groups, we analyzed 10 clinically-relevant gastric cancer therapeutics from the GDSC database (v2.0). Using the pRRophetic R package (35), we computed and compared half-maximal inhibitory concentrations (IC50) across risk strata, enabling systematic assessment of chemotherapy response patterns.

### 2.11 Immunohistochemical analysis and RT-qPCR validation

We validated the expression profiles of the four diagnostic and nine prognostic genes in gastric cancer (GC) using the Human Protein Atlas (HPA; https://www.proteinatlas.org/). Furthermore, RT-qPCR was employed to examine gene expression in clinical samples, which included five GC tissues and five matched adjacent non-tumor tissues. Total RNA was extracted with TRIzol reagent, and its purity and concentration were measured using a NanoPhotometer N50. cDNA was synthesized from the extracted RNA with the SweScript First Strand cDNA Synthesis Kit (Servicebio, China). Quantitative PCR was performed using the 2× Universal Blue SYBR Green qPCR Master Mix (Servicebio, China) in accordance with the manufacturer’s protocol. The 2^−ΔΔCt^ method was applied to calculate relative gene expression levels, using GAPDH as the endogenous control. All primer sequences were synthesized by Tsingke (Beijing, China) and are listed in **Supplementary Table 3**.

## 3 Results

### 3.1 Overlap analysis of genes profiled from bulk data

The PAN gene set was derived by integrating PAN genes from Reactome, AmiGO 2, and KEGG, with overlapping genes removed to obtain a non-redundant, high-confidence signature. Key apoptotic regulators included BAD, BIRC3, CASP8, CFLAR, E2F1, FOXO3, FOXO4, BCL2L1, BCL2, BBC3, AVEN, BCL6, JUN, HDAC9, IRF9, TFDP1, TNFRSF10A, TRAF2. pyroptosis-related genes were identified as CASP8, CARD8, GSDMA, GZMB, IFI27, IRGM, NFKBIA, NINJ1, OTULIN, PANX. Necroptosis-associated factors such as BIRC3, CASP8, CFLAR, NFKBIA, TNFRSF10A, TRAF2, BCL2, BCL2L1 and USP25 were also included. Moreover, several genes participated simultaneously in PAN, underscoring their critical role in PAN regulation (**Supplementary Table 2**).

### 3.2 Machine learning-based identification of molecular subgroups and predictive gene signatures in gastric cancer

**Figure 2:**
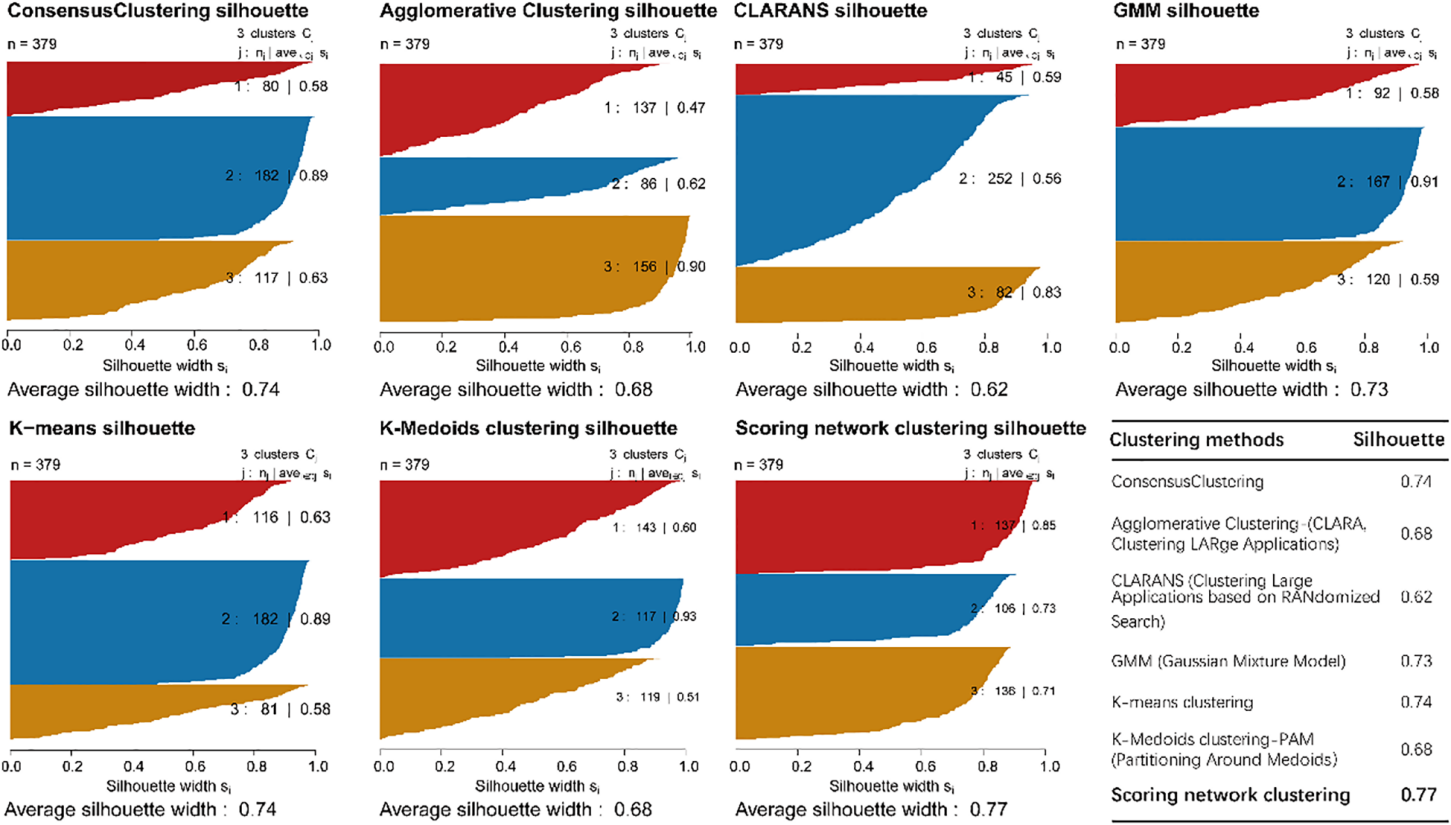
The sample similarity of each subtype was assessed by calculating the Silhouette score and comparative analysis of clustering performance.

We identified molecular subgroups by employing a voting-based scoring network that integrates five clustering algorithms—K-Means, GMM, CLARANS, Agglomerative Clustering, and K-Medoids—thereby enhancing the robustness and consistency of subtype classification. We independently identified three subtypes from five clustering algorithms, and the number of subtypes was determined by comprehensively referring to the cluster prediction silhouette score and previous research experience (**Figure 2**). Through comparative analysis with established clustering methodologies including Consensus Clustering, Agglomerative Clustering (CLARA), CLARANS, Gaussian Mixture Models (GMM), K-means clustering and K-Medoids clustering (PAM), our scoring network clustering algorithm demonstrated superior performance with the highest clustering score, confirming its effectiveness as a clustering approach (**Figure 2**).

**Figure 3:**
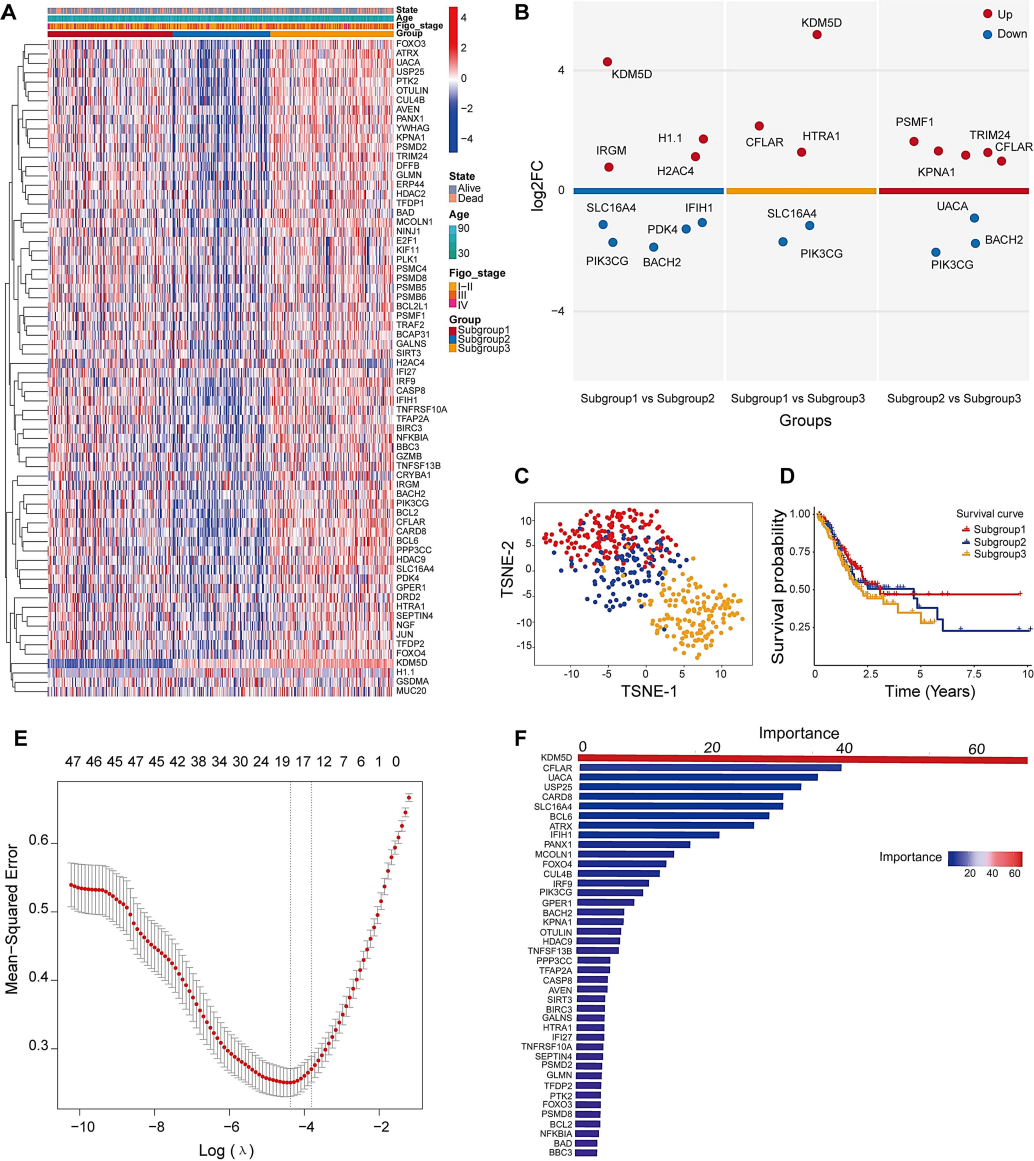
Identification of three distinct subtypes and key clustering genes in DEGs. **(A)** Heatmap analysis of PAN-related gene clusters and their clinicopathological correlations (Stage I-II: early cancer, curable; Stage III: Locally advanced cancer; Stage IV: spread widely or metastasized). **(B)** Volcano plots for multiple groups were drawn to show the differentially expressed genes in tumor subtypes tissue, up is high expression, down is low expression. **(C)** t-SNE dimensionality reduction analysis. **(D)** Survival analysis of molecular clusters. **(E)** Cross-validation curve of MSE versus log(λ) in LASSO regression. Dashed lines indicate the λ with minimum MSE and the λ within one standard error. **(F)** The RF importance score of these different expression genes among three subtypes.

The heatmap displays gene expression profiles across samples, where rows correspond to genes and columns to individual samples. A blue-to-red color gradient indicates expression levels, with blue denoting low and red indicating high expression. The dendrogram on the left represents hierarchical clustering of samples based on gene expression, revealing distinct clusters potentially corresponding to different molecular subgroups (**Figure 3A**). The expression patterns and predictive efficiency of these genes were further evaluated in the training set. KDM5D showed significant upregulation in Subgroup 1 compared to both Subgroup 2 and Subgroup 3, whereas SLC16A4 was downregulated. Additionally, CFLAR expression was elevated in Subgroup 2 relative to Subgroup 3, while UACA exhibited downregulation (**Figure 3B**). Based on gene expression patterns, the t-SNE plot illustrates three distinct subgroups, with samples represented as colored dots (Subgroup 1: red, Subgroup 2: blue, Subgroup 3: orange). The clear separation among subgroups suggests distinct molecular characteristics (**Figure 3C**). Kaplan-Meier survival curves for the three subgroups over a 10-year period show differences in survival probabilities. Despite clear molecular distinctions between subgroups, Kaplan-Meier analysis demonstrated no statistically significant survival differences (**Figure 3D**). To identify robust prognosis biomarkers, we implemented a dual machine learning approach combining LASSO regression (**Figure 3E**) for feature selection and random forest analysis (**Figure 3F**) for non-linear feature importance assessment. Through intersection analysis of both algorithms, we identified eight consensus diagnostic genes (KDM5D, CFLAR, UACA, SLC16A4, IFIH1, PIK3CG, TNFSF13B and PDK4) that demonstrated consistent predictive value across both methodologies.

### 3.3 Classification module using multi-scale cross-attention

**Figure 4:**
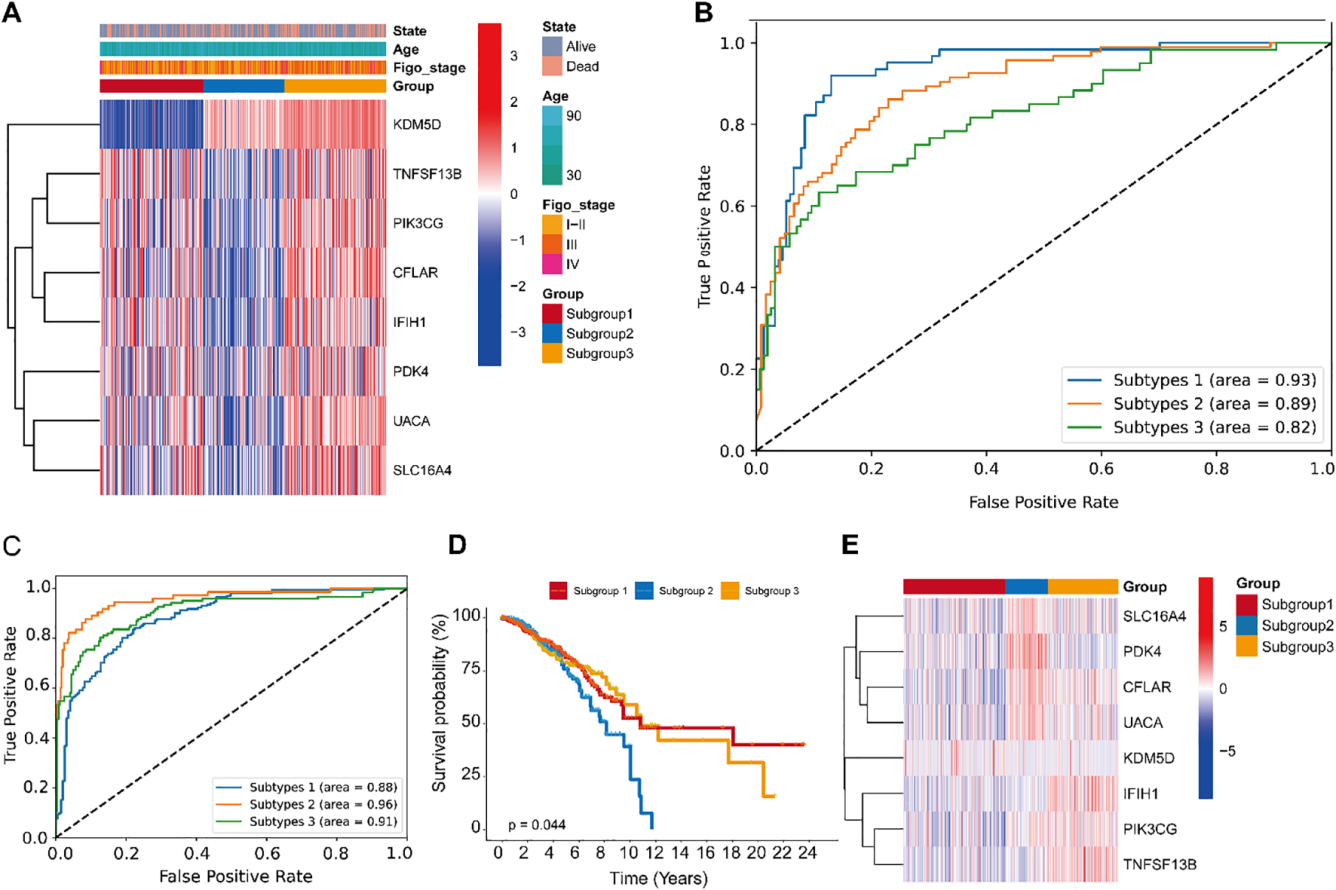
Performance evaluation of the CNN+BiLSTM classification module with multi-scale cross-attention in gastric cancer, and its scalability in the TCGA-BRCA cohort. **(A)** Distribution of input sample features used for model training and evaluation in gastric cancer. **(B)** ROC Curves of the CNN+BiLSTM model with multi-scale cross-attention. **(C)** The receiver operating characteristic curves (ROCs) of CNN+ BiLSTM classifier. **(D)** Kaplan Meier plot of the three subgroups identified by the classifier. **(E)** Expression patterns of the 10-gene profile are displayed, with predicted subgroup labels (generated by the CNN+BiLSTM classifier) annotated in the upper bar.

**Figure 4A** illustrates the expression profiles of feature genes across samples of different molecular subtypes. Within the heatmap, gene expression is displayed with genes on the y-axis and samples on the x-axis. The color coding reflects a variety of clinical and molecular characteristics, including survival status (alive or deceased), patient age, histological stage, sample grouping, and the expression levels of specific genes. The Receiver Operating Characteristic (ROC) curves in **Figure 4B** reveal the model’s excellent classification performance in distinguishing between different molecular subtypes, with the Area Under the Curve (AUC) values close to 1, indicating a high level of diagnostic accuracy. The accuracy rates (Train-Acc and Test-Acc) of different model variants on the training and testing datasets, respectively (**Supplementary Table 1**). These model variants are part of an ablation study conducted on the Multi-scale Cross-attention classification model. Ablation study is a model evaluation method that systematically removes or replaces certain parts of the model to understand the impact of these parts on the overall performance. The results from the ablation study indicate that the model combining CNN+BiLSTM performs the best on the test dataset, suggesting that considering both forward and backward sequence information along with convolutional feature extraction are key factors in enhancing the model’s performance within the Multi-scale Cross-attention classification model framework.

### 3.4 Generalization of the CNN+BiLSTM classification model

To comprehensively evaluate the model’s performance, we further tested its generalizability on two independent external datasets. Receiver Operating Characteristic (ROC) curves for different subtypes demonstrated robust classification performance, with Area Under the Curve (AUC) values of 0.88, 0.96, and 0.93, respectively. These high AUC values indicate that the model effectively distinguishes among the subtypes (**Figure 4C**). Kaplan-Meier survival curves revealed significant differences in survival outcomes among the subtypes, suggesting that the model can accurately stratify patients based on prognosis (**Figure 4D**). A heatmap of the feature gene expression profiles across subtypes illustrated distinct molecular signatures for each group. These patterns facilitate the identification of subtype-specific feature genes and provide insights into the underlying biological heterogeneity (**Figure 4E**). Collectively, these results demonstrate that the model possesses strong classification capability across different breast cancer subtypes, can effectively distinguish prognostic differences, and identify relevant molecular features. These findings offer a valuable foundation for further investigations into the molecular mechanisms of breast cancer and the development of personalized therapeutic strategies.

The classification results of the model on the CESC dataset comprehensively demonstrate its generalization performance, as reflected by training and testing accuracy, loss curves, ROC curves, survival analysis, and feature gene expression heatmaps. The model exhibits strong performance in distinguishing between different subtypes and accurately identifying subtype-specific feature genes. However, Kaplan-Meier survival analysis reveals no statistically differences among the subtypes, indicating potential limitations of the model in prognostic prediction. Despite this, the findings provide important evidence for further exploration of the molecular mechanisms of cervical squamous cell carcinoma and support the development of personalized treatment strategies (**Supplementary Figure 1**).

### 3.5 Validation optimal genes in scRNA transcriptome and spatial transcriptome

**Figure 5:**
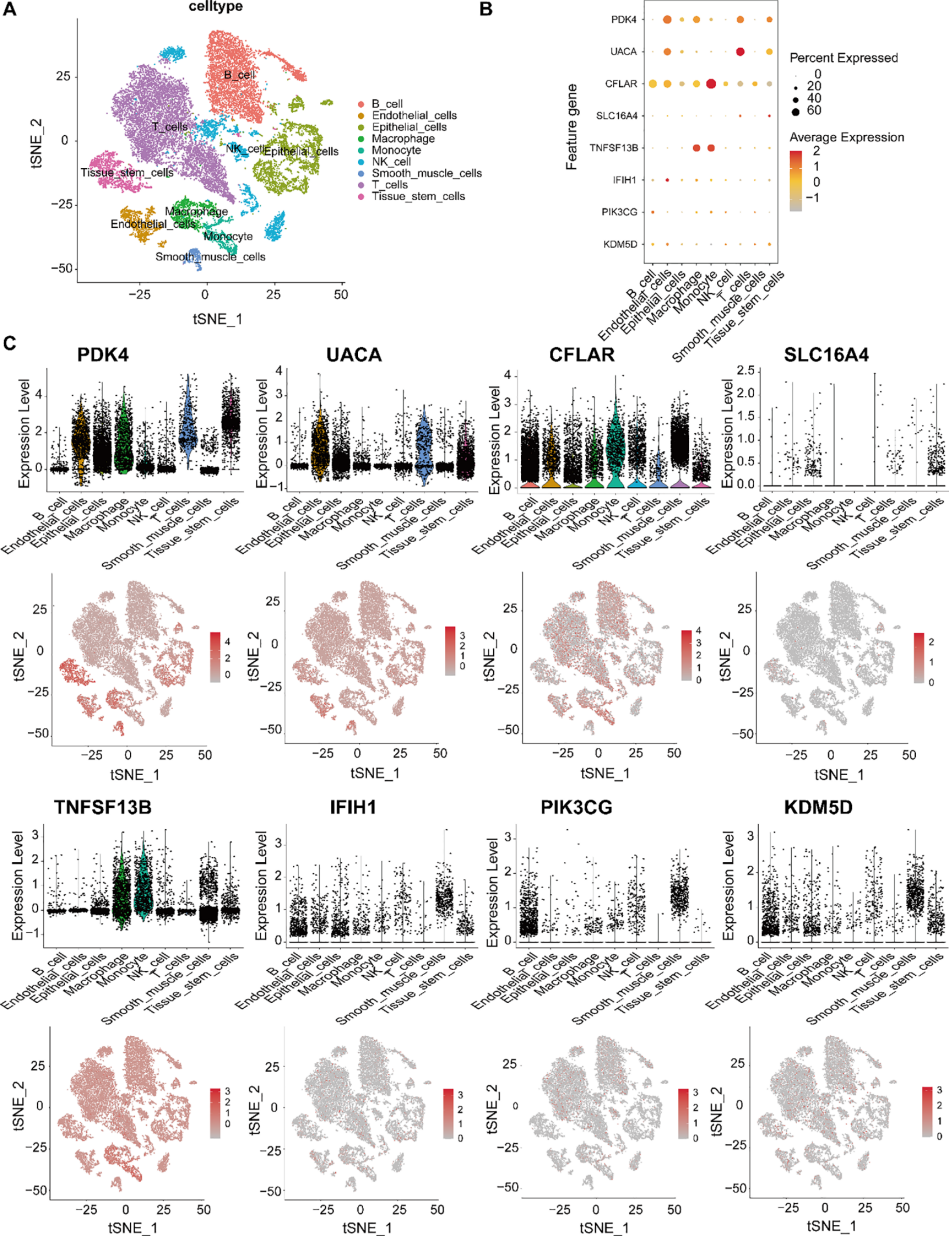
Single-cell transcriptomic profiling of candidate feature genes in gastric cancer. **(A)** t-SNE visualization of major gastric tumor microenvironment cell types: epithelial cells, T cells, B cells, NK cells, monocytes, macrophages, endothelial cells, and tissue stem cells. **(B)** Feature gene expression patterns across cell types, showing PDK4, UACA, CFLAR, SLC16A4, TNFSF13B, IFIH1, PIK3CG and KDM5D. Dot size indicates expression prevalence; color gradient represents expression intensity. **(C)** Violin plots and t-SNE feature maps showing the expression distribution of each gene at the single-cell level, revealing distinct cell-type-specific expression patterns and potential functional roles in gastric cancer.

To investigate the cellular context of candidate feature genes in gastric cancer, we analyzed their expression patterns using scRNA-seq data. The t-SNE plot (**Figure 5A**) revealed distinct clusters corresponding to various cell types within the tumor microenvironment, including epithelial and immune cell populations. As shown in the dot plot (**Figure 5B**), PDK4, UACA and CFLAR showed predominant expression in endothelial cells, while CFLAR and TNFSF13B were highly expressed in macrophages and monocytes. Notably, UACA and PDK4 demonstrated significant expression in immune cells (particularly T cells), suggesting potential immunomodulatory functions. In contrast, SLC16A4, IFIH1, PIK3CG and KDM5D exhibited relatively low and restricted expression patterns. Violin plots and t-SNE expression maps (**Figure 5C**) further validated these cell-type specific expression profiles, underscoring their potential roles in gastric cancer heterogeneity and immune microenvironment remodeling. The analysis reveals important insights into the cell-specific functions of candidate biomarkers, potentially guiding precision therapy development.

**Figure 6:**
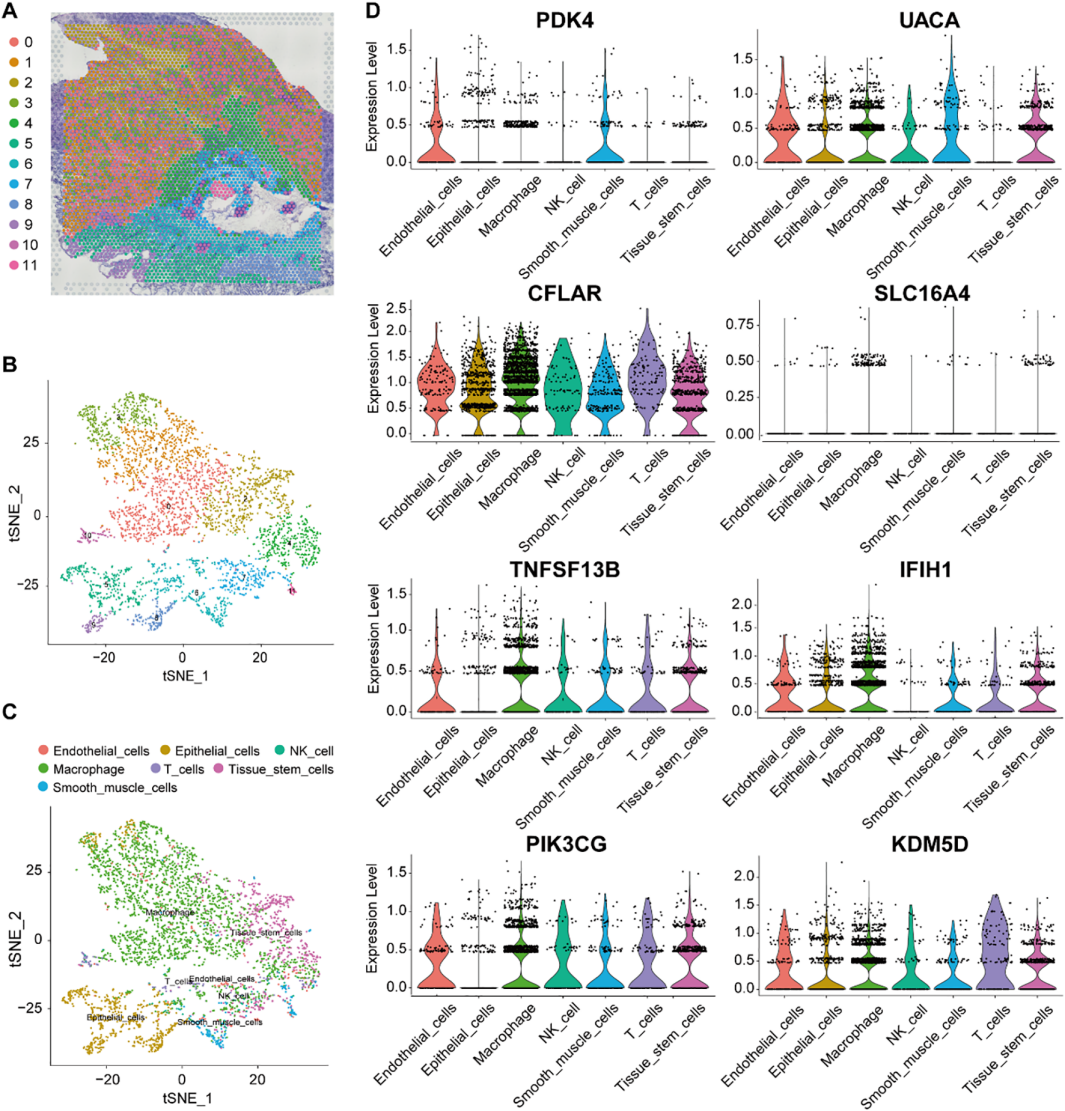
Validation marker genes in spatial transcriptomics data. **(A, B)** t-SNE projection of spatially resolved spot clusters in a gastric cancer patient sample. **(C)** tSNE visualization of the main cell type for each spot. **(D)** The distribution of PDK4, UACA, CFLAR, SLC16A4, TNFSF13B, IFIH1, PIK3CG and KDM5D expression across different cells types.

To characterize the spatial transcriptomic landscape of gastric cancer, we analyzed spatial transcriptomics data from gastric cancer samples (GSE251950, GEO database). Pathologist-validated annotations confirmed spatial correlations between programmed cell death and malignant tumor regions. Louvain clustering based on spatial transcriptomic profiles identified twelve distinct cellular clusters (**Figures 6A, B**). Integration with scRNA-seq data enabled deconvolution of seven clusters, revealing their cellular composition. Clusters 3, 5, 8 and 9 showed predominant localization in epithelial-rich regions, while macrophage-enriched clusters (0, 1, and 4) and other immune cell populations demonstrated distinct spatial distributions (**Figure 6C**). Expression mapping of the eight feature genes across major cell types revealed: macrophage and NK cells were primarily localized in cluster 7, with T cells concentrated in cluster 6 (**Figure 6C**). Genes including UACA and CFLAR exhibited elevated expression in epithelial cells, macrophages and endothelial cells, suggesting involvement in tumor-immune crosstalk. While SLC16A4 showed universally low expression, CFLAR demonstrated selective enrichment in T cells and NK cells. These spatially resolved expression patterns corroborated our scRNA-seq findings, further validating the cellular specificity and heterogeneity of the identified marker genes (**Figure 6D**, **Supplementary Figure 2**).

### 3.6 Endothelial cell crosstalk and pseudotime trajectory analysis

**Figure 7:**
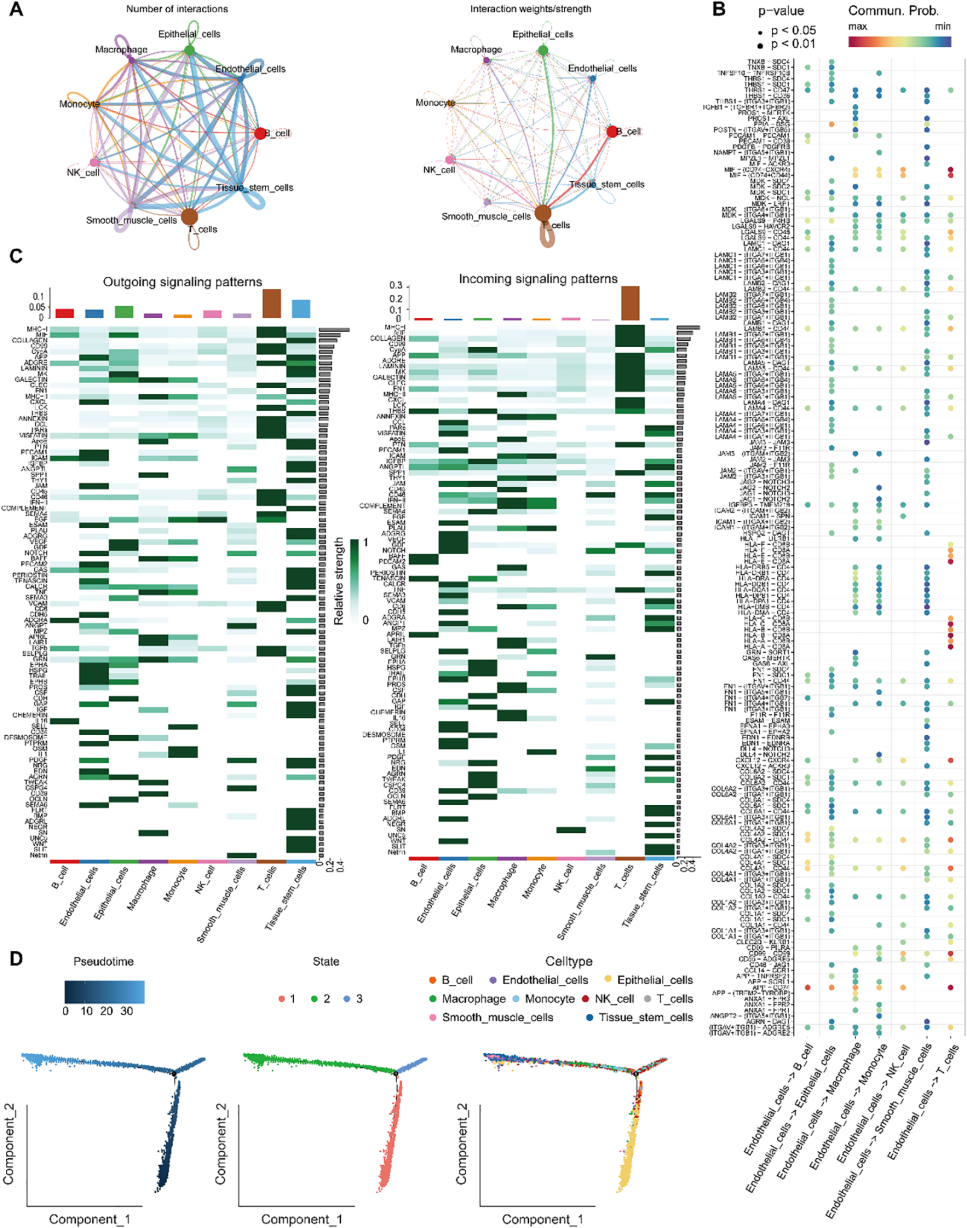
Analysis of endothelial cell communication and developmental trajectories. **(A)** Number and intensity of intercellular interactions between Endothelial_cells and other cell populations. **(B)** Bubble plot of ligand–receptor pairs from various cell types targeting Endothelial_cells. **(C)** Overview of signaling pathways mediating communication between Endothelial_cells and surrounding cell types. **(D)** Differentiation trajectory of Endothelial_cells, showing pseudotime progression and cluster distribution along the developmental timeline.

To elucidate the biological functions of endothelial cells in intercellular communication, we examined the extent and intensity of their connections with other cell types. Our findings indicated that endothelial cells are prominently engaged in a higher frequency of interactions, especially with immune cells and tissue stem cells, suggesting potential cooperative roles in these cellular networks (**Figure 7A**). Further analysis of ligand–receptor interactions revealed that endothelial cells communicate with other cell populations through specific signaling pathways (**Figure 7B**). In terms of signal reception, immune T cells more frequently interacted with endothelial cells via defined ligand–receptor pairs. Moreover, signaling pathways associated with endothelial cell communication, such as APP, CXCL, PECAM1, ICAM and JAM were more active in endothelial cells. Among the incoming signals, CCL, VISFATIN, PECAM1, ESAM and NOTCH were more prominently expressed in endothelial cells (**Figure 7C**).

Trajectory analysis of endothelial cells differentiation using Monocle 2 (**Figure 7D**) demonstrated that endothelial cells followed a differentiation path progressing from bottom to top along the pseudotime axis. During the initial phases of development, cells expressing key genes were relatively rare, but their prevalence significantly increased as development progressed. CytoTRACE analysis further delineated the developmental trajectory and origin of endothelial cells, indicating that the majority of endothelial cells were situated at more advanced stages of development.

### 3.7 Characterization of the tumor microenvironment via signature gene profiling

**Figure 8:**
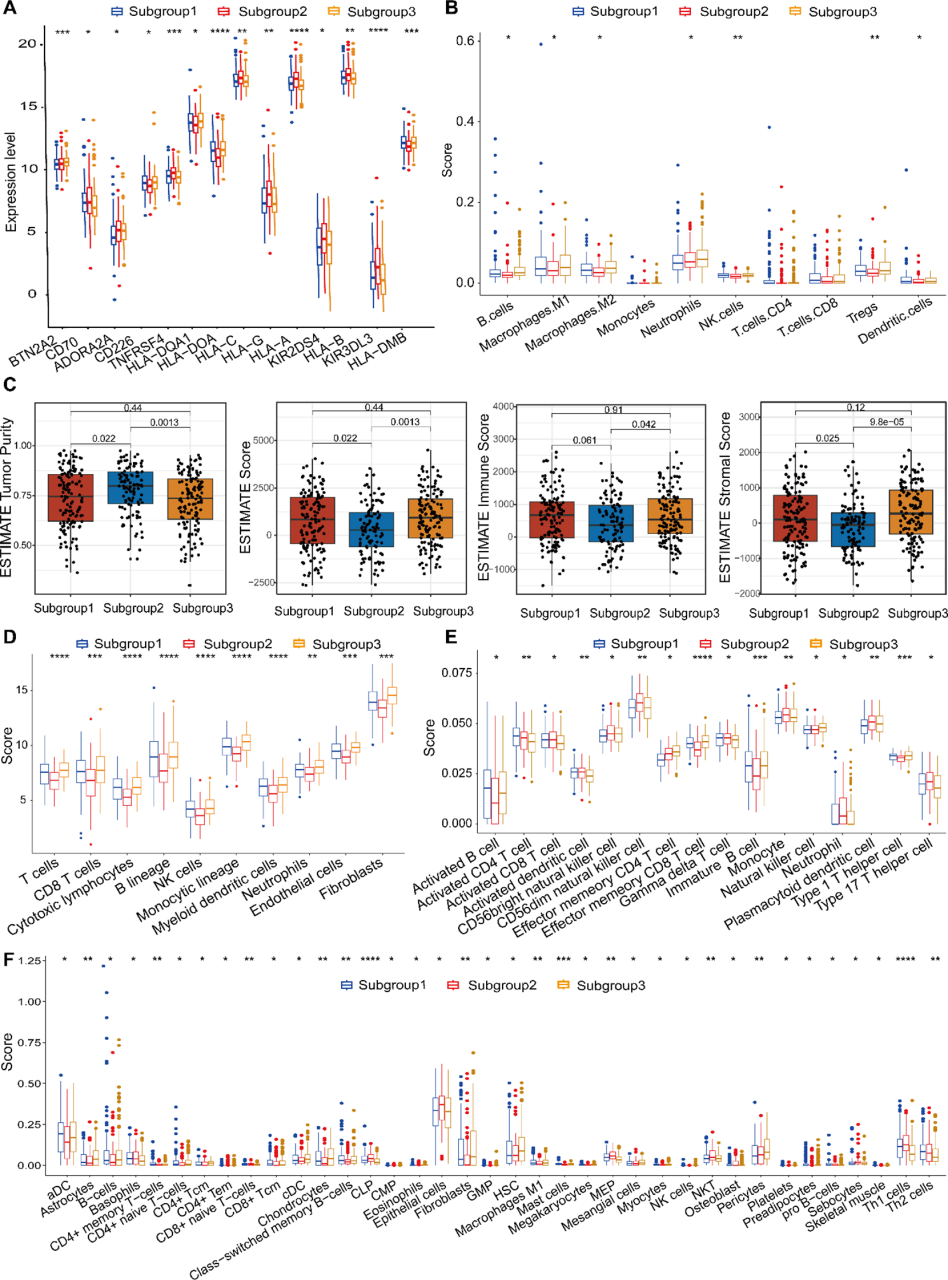
Tumor microenvironmental landscapes across the three molecular subtypes. **(A)** Comparative analysis of immune checkpoint gene expression across subtypes. **(B)** Immune cell composition inferred by quanTIseq: boxplots and heatmaps illustrating subtype-specific abundance patterns. **(C)** The ESTIMATE algorithm was employed to calculate tumor purity and stromal/immune infiltration scores. **(D)** Immune infiltration profiles derived from MCPcounter: boxplots comparing immune cell subsets between subtypes. **(E)** ssGSEA-based analysis of immune-related signatures across molecular subtypes. **(F)** xCell-derived immune cell abundance comparisons among the three subtypes. *p < 0.05, **p < 0.01, ***p < 0.001, ****p < 0.0001.

To further investigate the therapeutic relevance, the expression profiles of immune checkpoint genes were examined among the three molecular subtypes. As illustrated in **Figure 8A**, significant subtype-specific differences were observed in several immune checkpoint molecules, including HLA-DOA, HLA-A, KIR3DL3, BTN2A1, TNFRSF4, and HLA-DMB (P < 0.001); HLA-C, HLA-G, and HLA-B (P < 0.01); and CD70, ADORA2A, CD226, HLA-DQA1, and KIR2DS4 (P < 0.05). These results indicate that the molecular subtypes may differ in their immunoregulatory landscapes and potential responsiveness to immune checkpoint blockade, providing a rationale for subtype-specific immunotherapeutic strategies.

To comprehensively characterize the tumor immune microenvironment, we integrated multiple deconvolution approaches—quanTIseq, ESTIMATE, MCPcounter, ssGSEA, xCell, and CIBERSORTx—to evaluate immune infiltration and functional immune signatures. The quanTIseq method identified significant differences in infiltration levels for seven major immune cell subsets: B cells, Macrophages, NK cells, Tregs, Neutrophils and Dendritic cells (**Figure 8B**). Using the ESTIMATE algorithm, we assessed immune infiltration (stromal score, immune score, and ESTIMATE score) and tumor purity. Subgroup 1 and Subgroup 2, Subgroup 2 and Subgroup 3 significant differences in stromal or immune cell components were found between the two subgroups, indicating comparable tumor purity (**Figure 8C**). Subgroup 1 had lower immune cell infiltration levels compared to Subgroup 3, suggesting a little differences immune response. MCPcounter analysis demonstrated distinct immune infiltration profiles across subgroups. **Figure 8D** displayed highly significant differences among three subtypes (“P”<0.0001). Using ssGSEA, 16 types of infiltrating immune cells were evaluated. All listed cell types exhibited significant differences, indicating marked variations in their respective scores among the three subgroups. These findings further suggest substantial differences in the biological characteristics or functional states of these cell types across the subgroups, which may reflect distinct underlying biological processes or disease-associated states (**Figure 8E**). Using xCell, significant differences in infiltration scores were found for multiple cell types, including Epithelial cells, immature Dendritic cells (iDCs), and Megakaryocyte-Erythroid Progenitor cells (MEPs) exhibit the highest scores, whereas Activated Dendritic cells (aDCs), Fibroblasts, and Class-switched Memory B cells show the lowest in Subgroup 2. In contrast, Subgroup 3 is characterized by elevated scores in Astrocytes, Chondrocytes, Hematopoietic Stem Cells (HSCs), and Pericytes (**Figure 8F**). Across all analyses, immune infiltration levels were significantly different between the two molecular subtypes, with Subgroup 2 generally exhibiting higher immune cell infiltration.

The analysis indicates that TME plays a crucial part in influencing heterogeneous immune response and potentially guiding personalized immunotherapy strategies in gastric cancer.

### 3.8 Genomic mutation profiling

**Figure 9:**
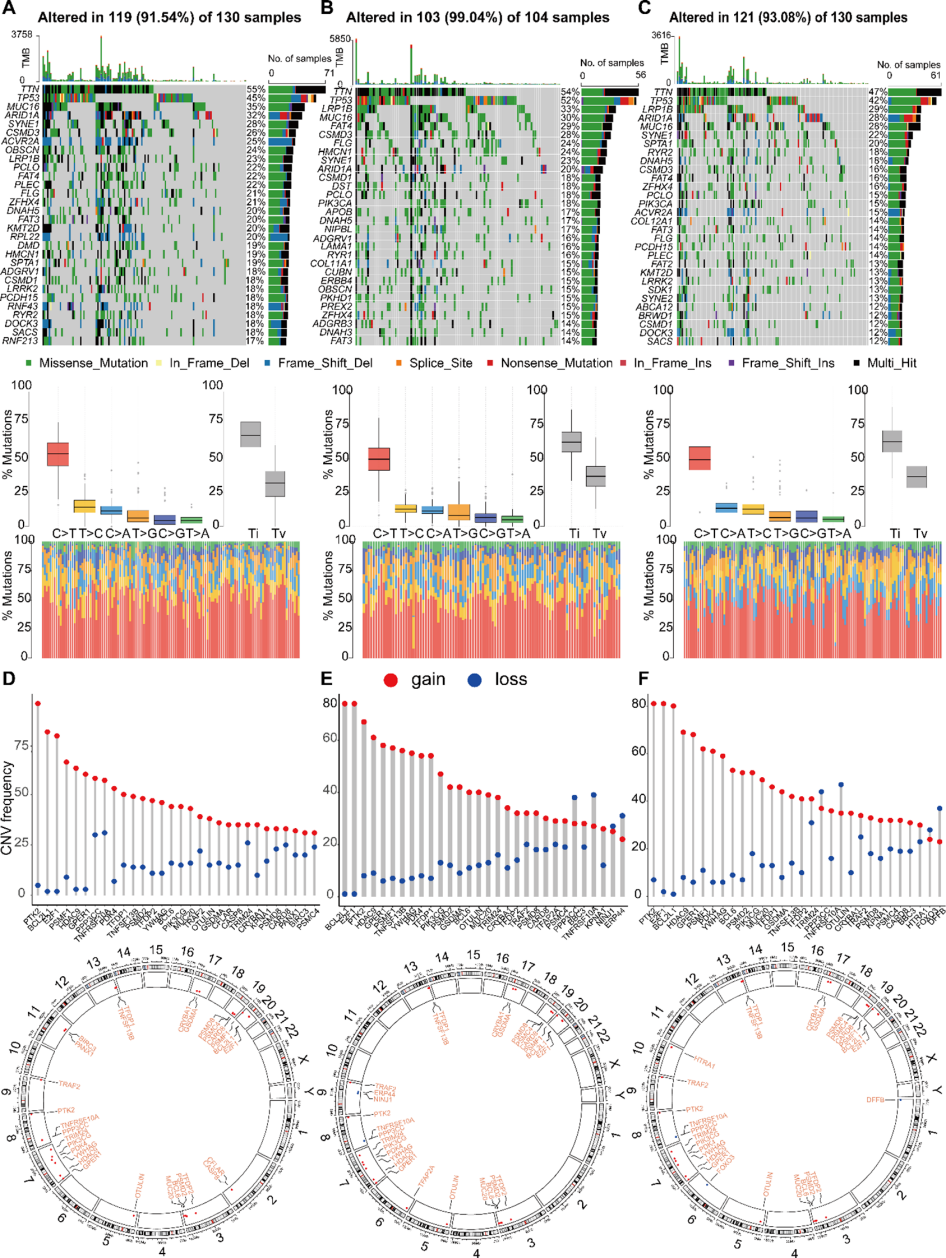
Landscape of single nucleotide variations (SNV) and CNV variation frequencies in three different groups. **(A-C)** The mutation frequency of top 30 genes, mutant spectrum characteristics and TMB distribution in three subgroups, respectively. **(D-F)** The CNV variation frequency of top 30 genes and the location on 23 chromosomes in subgroup 1, 2 and 3, respectively.

The efficacy of immunotherapy is influenced by multiple factors, including the infiltration patterns of immune cells in the TME and the mutational landscape of tumors. We hypothesized that the distinct molecular subtypes may exhibit differential tumor progression patterns and varied responses to immunotherapy. Since somatic mutations can generate neoantigens that enhance tumor-specific immune recognition, they represent promising targets for personalized immunotherapeutic strategies  (36). To explore this, we analyzed the somatic mutation profiles in gastric cancer patients to identify potential neoantigen sources. **Figures 9A-C** illustrates the top 30 genes exhibiting the most frequent mutations across the cohorts. Among them, ACVR2A, PLEC, ARID1A, ZFHX4, FAT3, and LRP1B showed significantly higher mutation frequencies in Subgroups 1 and 3. Notably, OBSCN mutations were exclusive to Subgroup 1. The mutation rate of KMT2D exhibited a decreasing trend from Subgroup 1 to 3 to 2, while the remaining genes displayed no statistically significant differences. Additionally, differentially mutated genes (DMGs) were enriched in Subgroups 1 and 3, indicating a cumulative impact of low-frequency variants, which may contribute to subtype-specific immunogenicity.

Among the three subtypes, genes such as PTK2, BCL2L11, and E2F1 exhibited high frequencies of copy number variations (CNVs). Notably, Subtype 1 showed elevated mutation frequencies on chromosomes 8 and 20, with copy number gains appearing to be more common than losses. Differences were observed in the distribution and frequency of CNV gains and losses among the subtypes. For instance, Subtype 2 was characterized by a unique alteration in NINJ1, along with increased frequencies of copy number losses in PPP3C, TNFRSF10A, and ERP44. In Subtype 3, the distinct alteration involved HTRA1, accompanied by elevated frequencies of copy number losses in PPP3C, TNFRSF10A, and DFFB. These differences may reflect the molecular characteristics of each subtype and provide insights into potential therapeutic targets (**Figures 9D-F**).

### 3.9 Immunohistochemical analysis and RT-qPCR validation diagnosis marker genes

**Figure 10:**
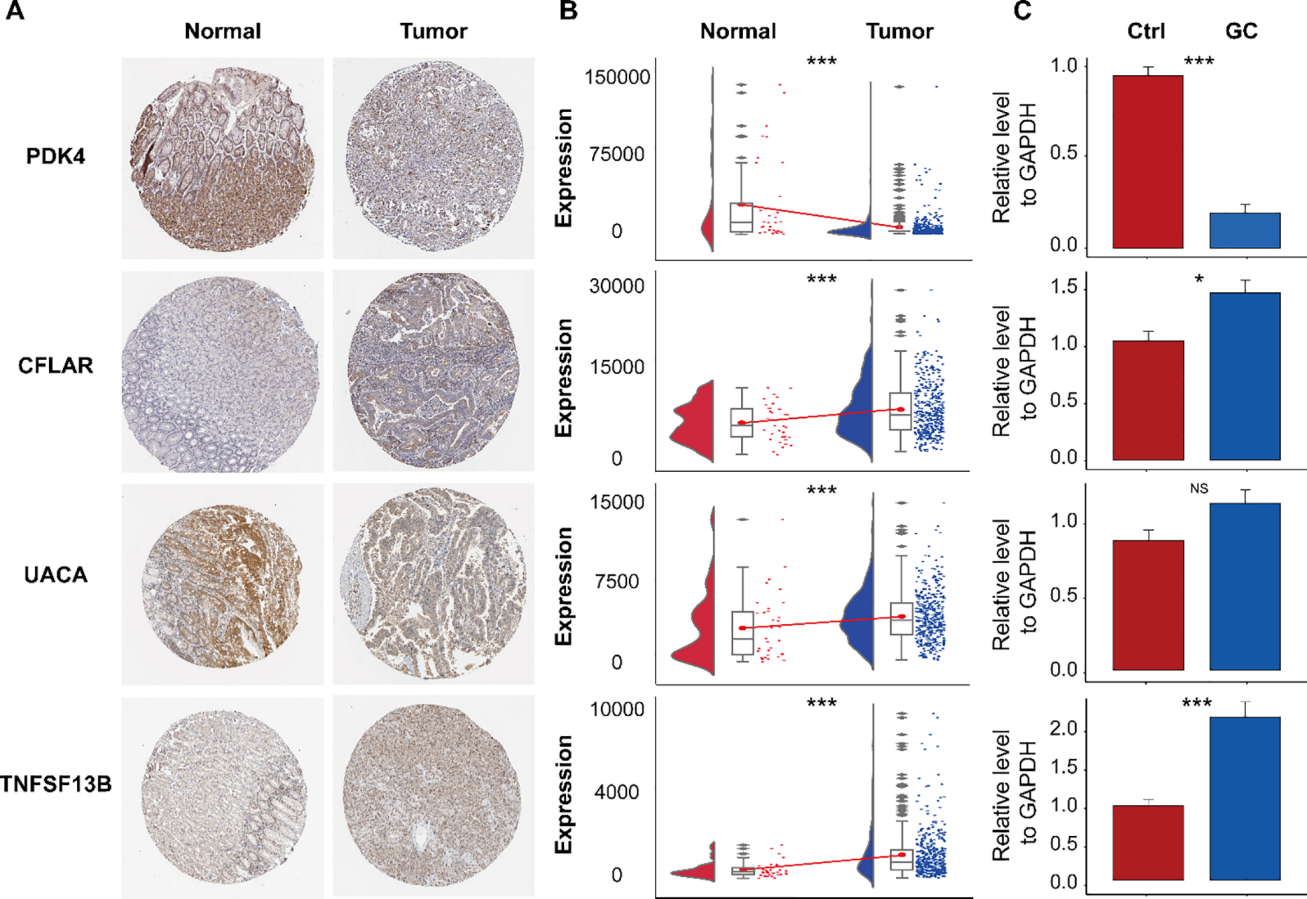
Immunohistochemical analysis of the HPA database and expression validation of marker genes. **(A)** Immunohistochemical staining of PDK4, CFLAR, UACA, and TNFSF13B in normal gastric tissues and gastric cancer (GC) tissues. **(B)** mRNA expression levels of the indicated genes in the TCGA-STAD dataset. **(C)** Validation of their expression by RT-qPCR. ns, not significant; *P< 0.05; ***P< 0.001.

We utilized immunohistochemical images sourced from the Human Protein Atlas database (HPA) to evaluate the protein expression levels of the four aforementioned marker genes. Our analysis compared protein expression in normal tissues and GC tissues to identify potential differences (**Figure 10**). The findings revealed that CFLAR and PDK4 exhibited significantly elevated protein expression in OC tissues compared to normal GC tissues (**Figure 10A**). However, PDK4 and UACA have low expression levels in GC tissues. TNFSF13B demonstrated expression with moderate staining intensity in GC tissues, whereas in normal gastric tissues, it exhibited lower levels of staining. In addition to UACA, the data study of the TCGA cohort found that it was consistent with the immunohistochemical trend (**Figure 10B**). Additionally, we assessed the expression levels of these genes in gastric cancer (GC) versus adjacent noncancerous tissues using RT-qPCR (**Figure 10C**). The results were consistent with the HPA database, showing that CFLAR and TNFSF13B were upregulated in GC tissues, whereas PDK4 was downregulated. In contrast, the expression of UACA showed no significant difference between GC and adjacent tissues, which was inconsistent with the Immunohistochemical results. This discrepancy may be attributed to the relatively small sample size in the Immunohistochemical results underscoring the need for further validation studies to confirm these observations.

### 3.10 Establishment and verification of a PAN gene-based prognostic model

**Figure 11:**
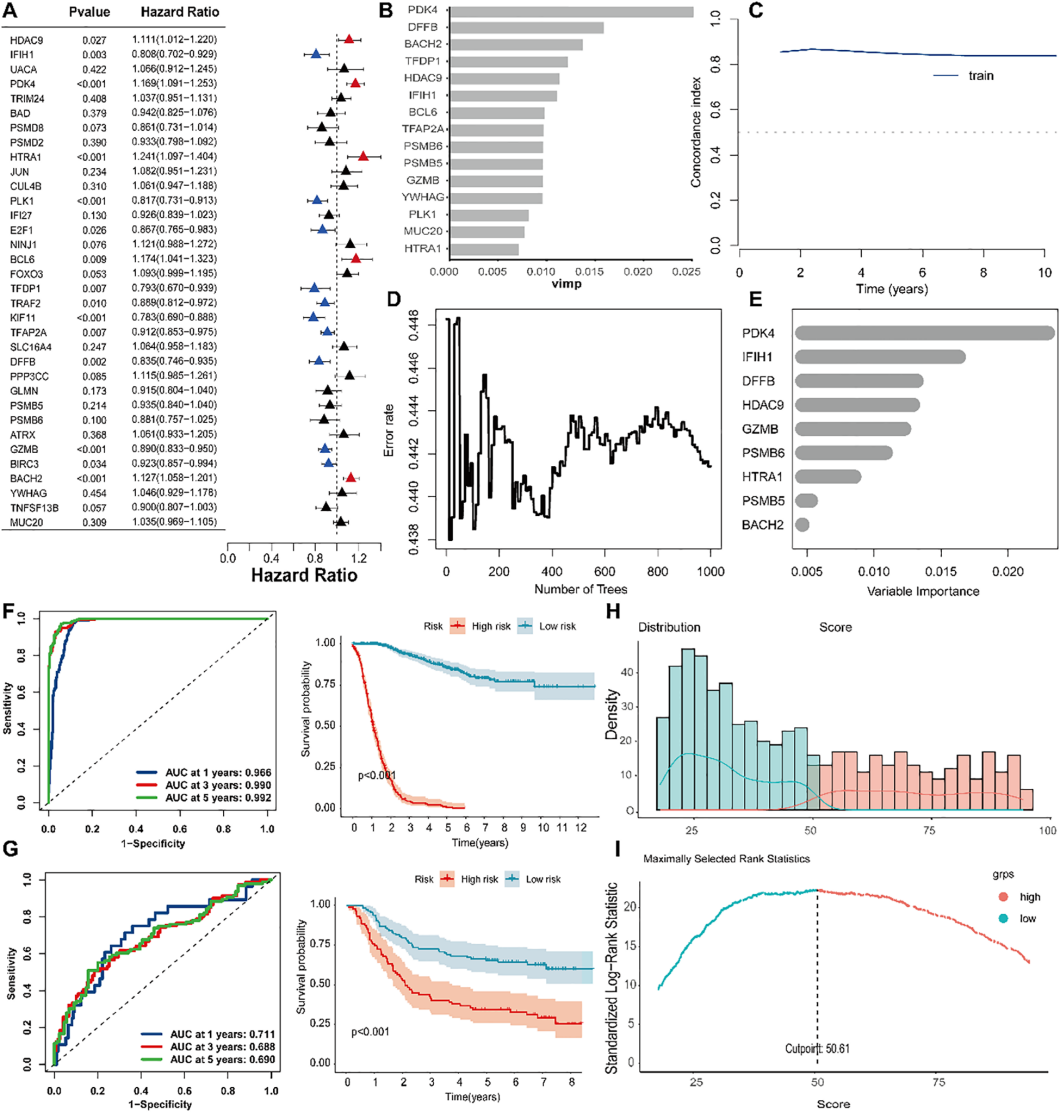
Construction and evaluation of the prognostic model based on the RSF algorithm. **(A)** Univariate Cox regression analysis identified survival-associated genes based on expression levels. **(B)** Gene importance ranking derived from the RSF model, with higher scores indicating greater contributions to predictive performance. **(C)** Time-dependent C-index evaluation of model performance. **(D)** The error rate of the RSF model decreased and eventually stabilized with an increasing number of decision trees, indicating strong model robustness and stability. **(E)** Top-ranked genes were identified as key variables with significant impacts on survival prediction. **(F)** In the training set, Kaplan-Meier analysis revealed significantly worse survival in high- versus low-risk patients. **(G)** Consistent survival trends were observed between risk groups in the validation cohort. **(H)** Risk score distribution clearly distinguished between high-risk and low-risk samples. **(I)** Optimal risk cutoff (50.61) derived from maximally selected rank statistics, maximizing intergroup survival difference.

To develop a prognostic model incorporating PAN-related genes, we employed Cox regression analysis coupled with Random Survival Forest to optimize the selection of prognostic differentially expressed genes in gastric cancer. Univariate Cox regression identified genes significantly associated with overall survival (P < 0.05, HR ≠ 1), as shown in **Figure 11A**. Subsequently, RSF analysis ranked the top 15 survival-associated genes according to their importance scores, with higher values reflecting a greater contribution to predictive performance (**Figure 11B**). **Figure 11C** illustrates the time-dependent predictive accuracy of the RSF model, which maintained a high and stable concordance index (C-index), indicating strong prognostic capability. With increasing numbers of decision trees, we observed a consistent reduction in error rate that eventually reached a stable plateau (**Figure 11D**), demonstrating its robustness and stability. Among the top-ranked genes, PDK4, IFH1, DFFB, HDAC9, GZMB, PSMB6, HTRA1, PSMB5, and BACH2 emerged as key contributors to survival prediction (**Figure 11E**), and were selected for further model construction and validation. *f* (*t* ∣ x) = *f*_0_(*t*) × exp (*coef*_1_*gene* 1 +*coef*_2_*gene* 2 + ⋯+*coef*_9_*gene* 9), where *f* (*t* ∣ x) represents the risk function at time *t*, conditioned on the covariates *x*; *f*0(*t*) is the baseline risk function; *coef*_*n*_ is the coefficient of each predictive variable factor; and *gene*_n_ is the gene affecting survival.

Our prognostic model achieved outstanding discrimination (AUCs: 0.966-0.992 across 1–5 years) and effectively stratified patients into clinically distinct risk groups. Both primary and validation cohorts showed significantly reduced survival in high-risk patients (**Figures 11F, G**). The optimal risk cutoff (50.61) maximized survival separation between groups (**Figures 11H, I**), confirming robust predictive performance.

### 3.11 Drug sensitivity prediction

**Figure 12:**
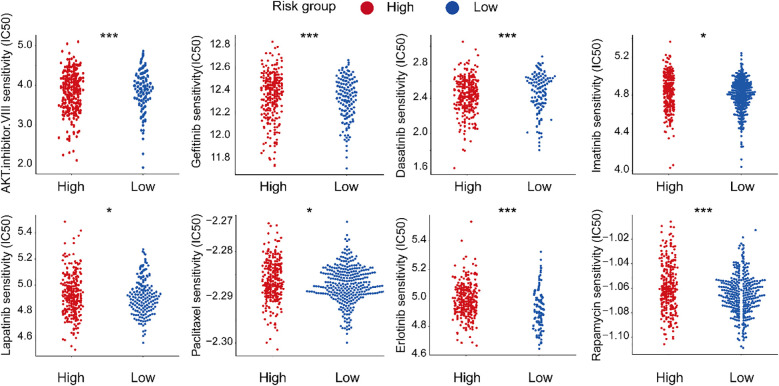
Drug sensitivity stratification in gastric cancer. *P< 0.05; ***P< 0.001.

To evaluate the clinical utility of our PAN-based model, we analyzed IC50 values of common anticancer drugs in gastric cancer samples. Eight drugs showed differential sensitivity between the higher and lower PAN scoring groups (**Figure 12**). The high-PANscore group exhibited greater sensitivity to AKT inhibitor VIII, Dasatinib, and Lapatinib (lower IC50), while demonstrating potential resistance to Gefitinib, Imatinib, Paclitaxel, Etoposide, and Rapamycin (higher IC50). These results suggest our model may guide personalized therapy selection in gastric cancer patients.

### 3.12 Protein expression and RT-qPCR detection of prognostic signatures

**Figure 13:**
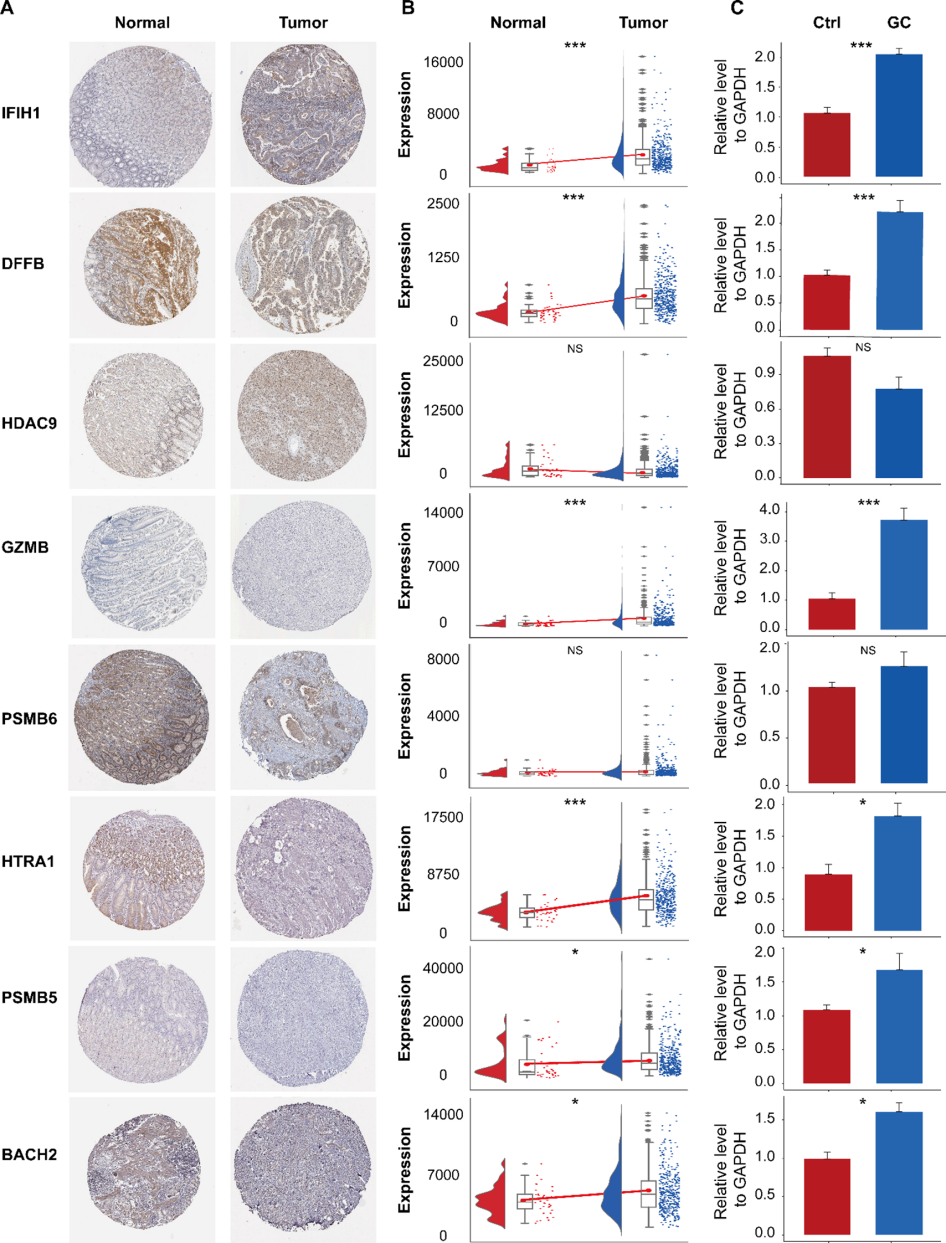
Immunohistochemical analysis of the HPA database and expression validation of prognostic genes. **(A)** Immunohistochemical staining of IFIH1, DFFB, HDAC9, GZMB, PSMB6, HTRA1, PSMB5, and BACH2 in normal gastric tissues and gastric cancer (GC) tissues. **(B)** Expression levels of these genes in the TCGA-STAD dataset. **(C)** Validation of their expression by RT-qPCR. ns, not significant; *P< 0.05; ***P< 0.001.

Analysis of HPA and TCGA-STAD data revealed differential expression patterns of IFIH1, DFFB, PSMB6, PSMB5, and BACH2. Specifically, DFFB and PSMB6 exhibited significantly higher expression in control samples, whereas GLP1R was upregulated in HCC tissues (**Figures 13A, B**). No significant differences were observed in HDAC9 and BACH2 expression levels between groups (**Figures 13A, B**). RT-qPCR validation confirmed these findings, demonstrating significant differences in gene expression between gastric cancer (GC) and control samples, consistent with the HPA and TCGA-STAD datasets (**Figure 13C**). However, HDAC9 and PSMB6 did not reach statistical significance (P > 0.05), which may be attributed to limited sample size.

## 4 Conclusions and discussion

In this study, we systematically investigated the interplay among three programmed cell death pathways to define a PAN-related molecular landscape in gastric cancer. While the three molecular subtypes defined by these genes did not show statistically significant differences in overall survival (**Figure 3D**), this finding is itself insightful. It highlights that the primary value of this molecular subtyping lies not in direct prognosis prediction, but in its power to resolve the profound heterogeneity of gastric cancer. These subtypes exhibited starkly distinct tumor microenvironment (TME) profiles, genomic alteration patterns, and predicted drug sensitivities (as shown in **Figures 8**, **9**). This stratification provided the essential biological context to identify the key genes and mechanisms that drive aggressive disease within this heterogeneous landscape. In other words, the subtyping uncovered the “source of heterogeneity”, which we then leveraged to build a refined and highly predictive tool.

The experimental validation of our computational findings, particularly the consistent upregulation of CFLAR and downregulation of PDK4 in gastric cancer tissues, provides a solid foundation for proposing their mechanistic roles (15). CFLAR (c-FLIP) is a critical anti-apoptotic regulator that competes with caspase-8 for binding to FADD, thereby inhibiting the extrinsic apoptosis pathway. Its significant overexpression in our GC cohorts suggests a potent mechanism by which tumor cells may evade this form of cell death. Intriguingly, CFLAR has also been implicated in modulating necroptosis. We hypothesize that in GC, CFLAR overexpression creates a cell death “rheostat,” preferentially shutting down apoptosis and potentially diverting cell fate towards other PAN modalities, thereby contributing to tumor survival and therapeutic resistance.

Conversely, the downregulation of PDK4 points to a profound metabolic reprogramming. PDK4 phosphorylates and inactivates the pyruvate dehydrogenase complex (PDH), thereby preventing the entry of pyruvate into the mitochondrial TCA cycle. Its suppression in GC suggests a shift towards enhanced mitochondrial oxidative phosphorylation. Since efficient mitochondrial function is linked to the generation of reactive oxygen species (ROS) and other signals that can trigger pyroptosis, we speculate that PDK4 downregulation may be an adaptive mechanism to reduce mitochondrial stress and avoid this inflammatory form of cell death (5, 9). This would allow the tumor to grow without eliciting a robust immune response, aligning with the immune-evasive phenotypes we observed in high-risk groups.

Building on these mechanistic insights from individual genes, we integrated them into the PANscore. The model’s outstanding prognostic accuracy (5-year AUC of 0.992) demonstrates that the collective derangement of these interconnected cell death and metabolic pathways is a powerful determinant of patient outcomes. The PANscore, therefore, moves beyond simple subtype classification to quantify a tumor’s functional state of “PAN resistance.” This state is characterized not by the absence of immune cells, but by an inability to effectively execute immunogenic cell death, leading to an inflamed yet immunosuppressed TME, as our data show (11, 12, 37). This aligns with emerging evidence that machine learning can capture such latent biological variation in high-dimensional transcriptomic data (17).

This mechanistic framework directly informs the therapeutic vulnerabilities we identified. The efficacy of AKT inhibitors in high-PANscore patients may lie in AKT’s known role in regulating both metabolism and cell survival, potentially forcing a lethal rewiring in these already dysregulated tumors (1). Similarly, the potential resistance to Gefitinib could be explained by the robust anti-apoptotic shield provided by high CFLAR and other PANscore components, underscoring the need for combination therapies that co-target these resistance mechanisms. Our drug sensitivity analysis thus supports the development of stratified therapeutic approaches based on this quantitative risk assessment (1, 44).

Our study provides a robust multi-omics landscape of PAN in GC, which directly points to the testable mechanistic hypotheses outlined above (2, 7, 10). The key limitation is the lack of functional validation for these hypotheses. Specifically, future work must experimentally determine whether CFLAR indeed functions as the proposed rheostat controlling the apoptosis-necroptosis switch in GC cells, and whether restoring PDK4 expression can sensitize tumors to pyroptosis-inducing therapies. Employing genetic perturbations *in vitro* and *in vivo* will be crucial to establish causality within the PAN network and to translate our computational insights into targeted therapeutic strategies (3, 4, 6).

## Data Availability

The original contributions presented in the study are included in the article/**Supplementary Material**. Further inquiries can be directed to the corresponding author.
