# Supplementary material for: Deep learning and multi-omics reveal programmed cell death-associated diagnostic signatures and prognostic biomarkers in gastric cancer
Source: Front Immunol. 2025 Nov 10;16:1690200. doi: 10.3389/fimmu.2025.1690200 (PMC12640992; doi:10.3389/fimmu.2025.1690200)
Supplement: Supplementary file 1 [file Image1.pdf]

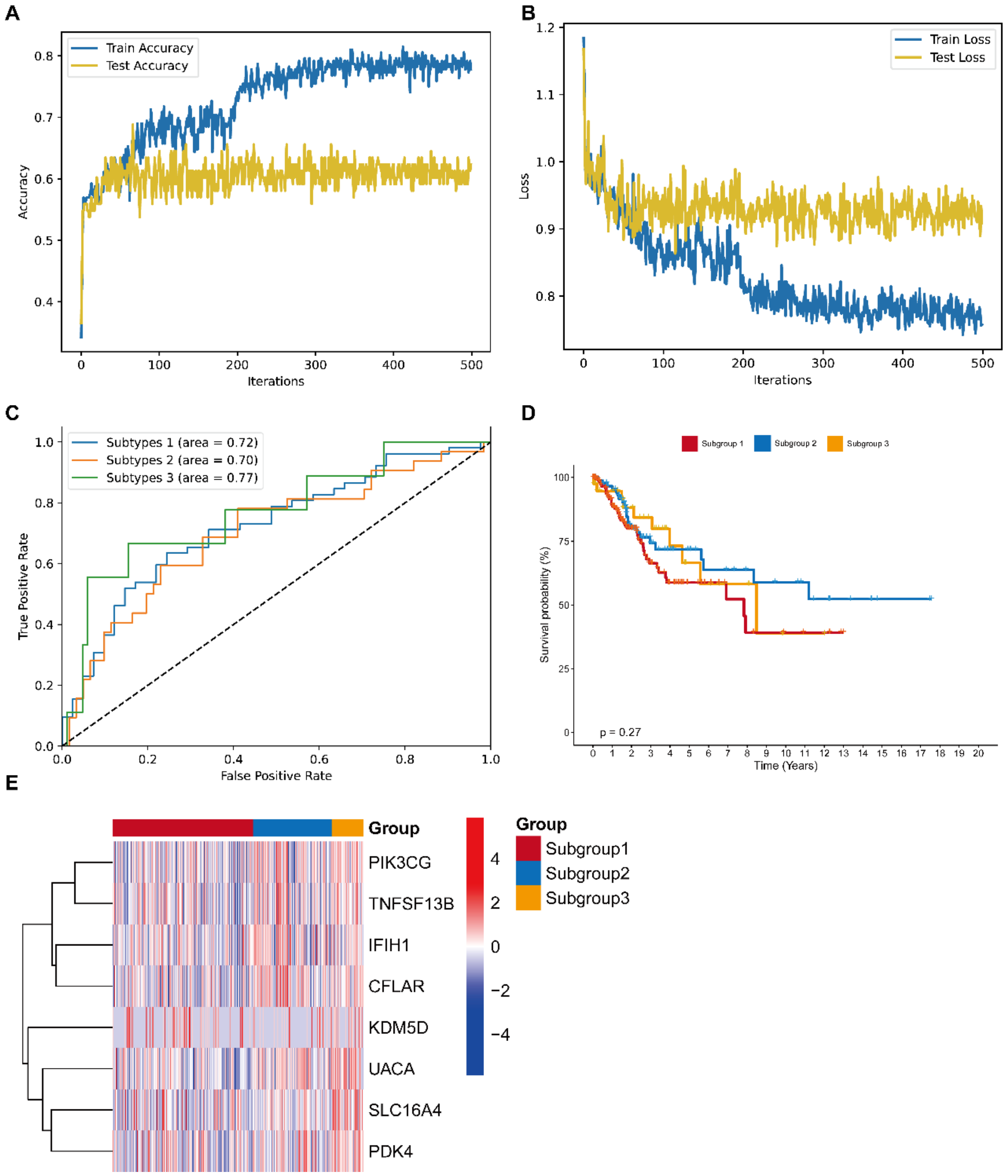

Figure S1 The scalability of CNN+BiLSTM classifier in TCGA-CESC cohorts. **(A, B, C)** The change of the Accuracy, Loss value with increasing iterations, the receiver operating characteristic curves (ROCs) of CNN+ BiLSTM classifier. **(D)** Kaplan Meier plot of the three subgroups. **(E)** Expression patterns of the 10-gene signature in the TCGA-CESC cohort are displayed, with predicted subgroup labels (generated by the CNN+BiLSTM classifier) annotated in the upper bar.

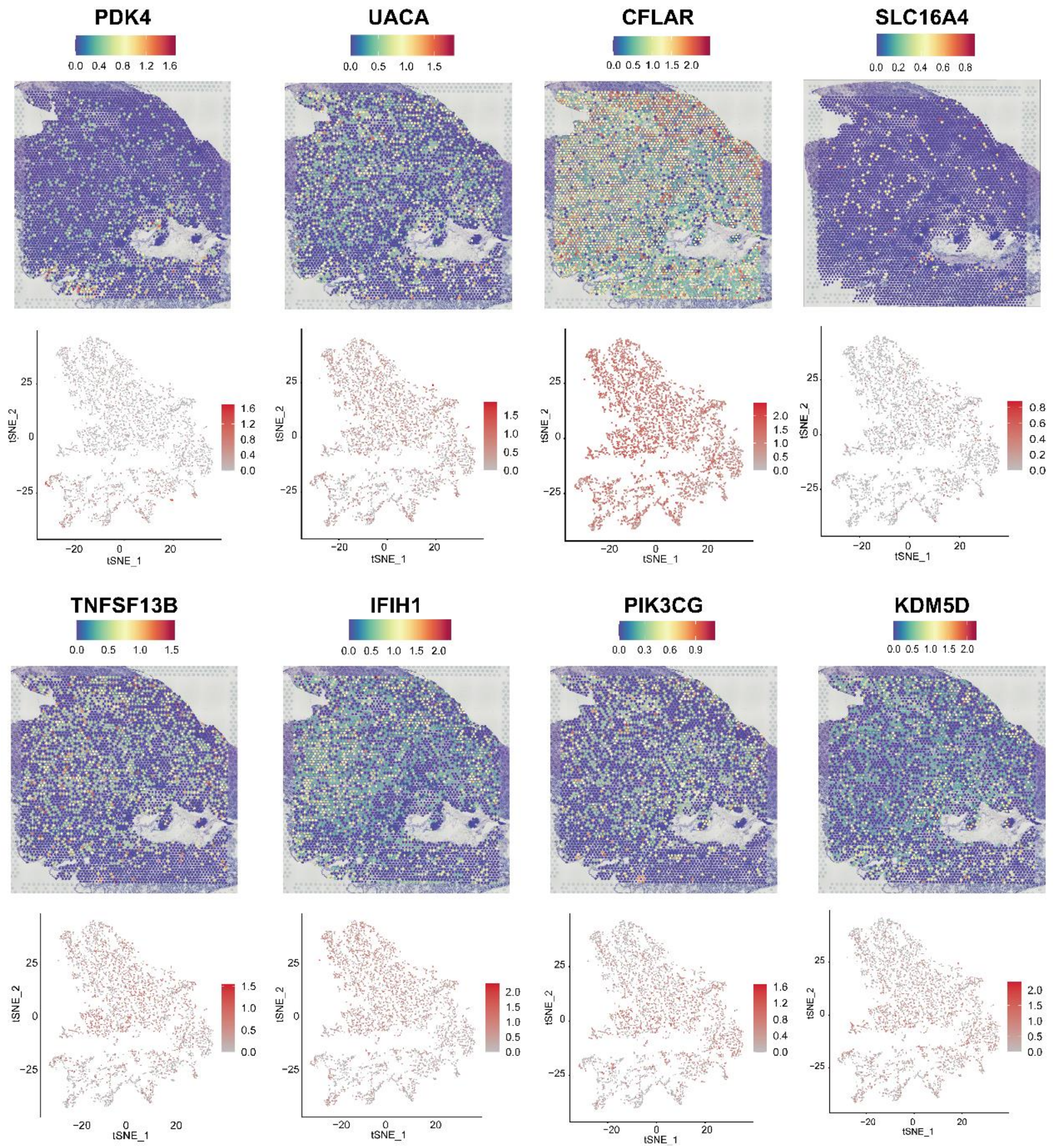

Figure S2 Projections and t-SNE visualizations of the expression patterns of gastric cancer marker genes.
